# Marker-free carotenoid-enriched rice generated through targeted gene insertion using CRISPR-Cas9

**DOI:** 10.1038/s41467-020-14981-y

**Published:** 2020-03-04

**Authors:** Oliver Xiaoou Dong, Shu Yu, Rashmi Jain, Nan Zhang, Phat Q. Duong, Corinne Butler, Yan Li, Anna Lipzen, Joel A. Martin, Kerrie W. Barry, Jeremy Schmutz, Li Tian, Pamela C. Ronald

**Affiliations:** 1 Department of Plant Pathology and the Genome Center, University of California, Davis, CA 95616 USA; 2 Innovative Genomics Institute, Berkeley, CA 94704 USA; 3 Feedstocks Division, The Joint Bioenergy Institute, Emeryville, CA 94608 USA; 4 Department of Plant Sciences, University of California, Davis, CA 95616 USA; 5 Department of Energy Joint Genome Institute, Berkeley, CA 94720 USA

**Keywords:** Metabolic engineering, Molecular engineering in plants, Plant breeding, Plant molecular biology

## Abstract

Targeted insertion of transgenes at pre-determined plant genomic safe harbors provides a desirable alternative to insertions at random sites achieved through conventional methods. Most existing cases of targeted gene insertion in plants have either relied on the presence of a selectable marker gene in the insertion cassette or occurred at low frequency with relatively small DNA fragments (<1.8 kb). Here, we report the use of an optimized CRISPR-Cas9-based method to achieve the targeted insertion of a 5.2 kb carotenoid biosynthesis cassette at two genomic safe harbors in rice. We obtain marker-free rice plants with high carotenoid content in the seeds and no detectable penalty in morphology or yield. Whole-genome sequencing reveals the absence of off-target mutations by Cas9 in the engineered plants. These results demonstrate targeted gene insertion of marker-free DNA in rice using CRISPR-Cas9 genome editing, and offer a promising strategy for genetic improvement of rice and other crops.

## Abstract (web summary)

Existing examples of targeted gene insertion in plants either rely on a selectable marker gene or result in short DNA inserts. Here, the authors use an optimized CRISPR-Cas9 method to insert a 5.2 kb carotenoid biosynthesis cassette into genomic safe harbors in rice, and obtain marker-free lines with high carotenoid content.

## Introduction

Conventional *Agrobacterium*- or particle bombardment-based plant transformation integrates transgenes at random locations in the plant genome, which can sometimes reduce yield in the resulting plants^1^. Genomic safe harbors (GSHs)^2^ are chromosomal regions that can accommodate transgenes without adverse effects on the host organism due to genome disruption. Targeted gene insertion at double-strand breaks (DSBs) in the GSHs provides a desirable alternative to conventional plant transformation methods^3^. Recent advances in genome editing technologies have enabled the induction of DSB at defined targets in a relatively simple manner, paving the way for targeted gene insertion in plants^4,5^.

CRISPR-Cas is by far the most widely used genome editing platform due to its efficacy, versatility, and simplicity^6^. The CRISPR-Cas system typically consists of a sequence-specific nuclease such as Cas9, and a guide RNA (gRNA), which mediates the recognition of a target sequence and cleavage at that site by the nuclease. Although efficient CRISPR-Cas-based tools for gene knockout in diverse plant species have been developed^7–10^, targeted gene insertion in plants by CRISPR-Cas has proved to be more challenging^11,12^. Most reported examples of targeted gene insertion by CRISPR-Cas in plants are dependent on chemical selection of the inserted cassette^13–19^. For this reason, a selectable marker gene is often included in the inserted cassette to enable selection with an herbicide. A disadvantage to this approach is that the marker gene takes up valuable space within the cassette and it is retained in subsequent generations together with the desired trait. Products obtained through such approaches often require additional regulatory approvals and can trigger public concern. The few cases of targeted insertion of marker-free DNA fragments in plants have been achieved with relatively small DNA fragments (ranging from 281 bp to 1.8 kb)^20–23^. The small size of the DNA insert restricts the amount of genetic information that can be introduced. In this study, we demonstrate the targeted insertion of a 5.2 kb marker-free DNA fragment at two GSHs in rice using CRISPR-Cas, and obtain homozygous carotenoid-enriched rice.

## Results

### Choosing gene insertion targets in a model rice variety

Rice (*Oryza sativa*) is a staple food crop for more than half of the world’s population. To identify GSHs in rice, we conducted a mutant screen by analyzing the morphological records and the whole-genome sequencing data of a fast-neutron rice mutant collection in a model *japonica* rice variety with a short generation time^24–26^. From this screen, we identified five mutants carrying homozygous insertions or translocations (Supplementary Table 1), which do not exhibit visible morphological changes compared with the parental genotype. We verified the homozygous mutations in these mutants by PCR using primers flanking the corresponding mutation sites (Supplementary Fig. 1). Because the mutations do not incur any visible change in morphology, these five intergenic mutation sites (A, B, C, D, and E) were chosen as the candidate GSHs (Supplementary Table 1).

Using the CRISPR-PLANT guide RNA design platform^27^, we selected seven specific sites near the five candidate GSHs in the Kitaake rice genome^28^, and designed gRNAs (A, B, C, D1, D2, E1, and E2) targeting each of these sites (Supplementary Fig. 2a). To experimentally determine the ability of Cas9 to induce DSBs at each of the seven targets in vivo, we performed a T7 Endonuclease 1 (T7E1) assay^29^ in rice protoplasts transiently expressing *Cas9* and each of the seven gRNA candidates. This assay quantifies the frequency of Cas9-induced mutations at each of the seven gRNAs targets, which reflects the efficiency of cleavage by Cas9 at these targets in vivo. Mutations occurred at targets A, B, and C at relatively high frequencies (Supplementary Fig. 2b), indicating that the gRNAs can target these sites.

### Constructing a maker-free carotenoid cassette for insertion

**Fig. 1:**
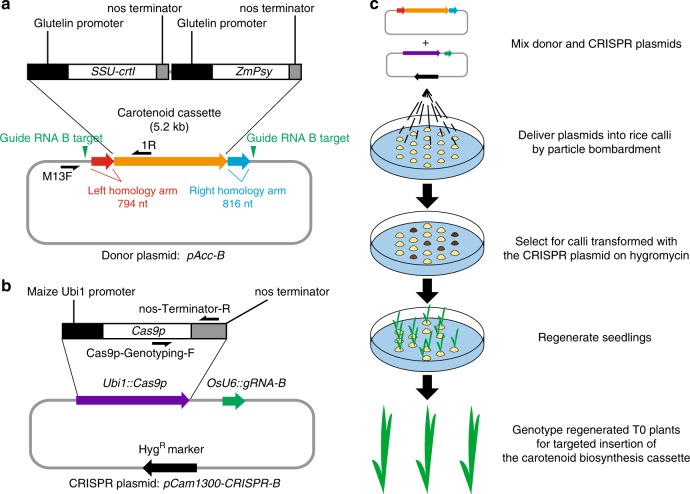
Scheme for targeted insertion of the carotenoid cassette. **a** Map of the donor plasmid *pAcc-B* with details of the carotenoid cassette (orange arrow). Red and blue arrows represent the homology arms. The two vertical green triangles mark the positions of the guide RNA B target sites. The nucleotide sequence of the donor plasmid is presented in Supplementary Data 1. Primers used to genotype the donor plasmid are marked on the map. **b** Map of the CRISPR plasmid *pCam1300-CRISPR-B*. Genes encoding *Cas9p*, *gRNA-B*, and hygromycin resistance (Hyg^R^) are represented by purple, green, and black arrows, respectively. The *Cas9p* module is shown in detail. Primers used to genotype *Cas9p* are marked on the map. **c**, Scheme for transformation, selection, and regeneration.

Because of the valuable socio-economic impact conferred by the Golden Rice 2 (GR2) cassette, its availability, and the clear phenotype it confers^30^, we chose to modify this cassette and use it as the donor DNA to assess the efficiency of marker-free targeted insertion in rice. Rice varieties carrying the Golden Rice 1 (GR1) and the GR2 cassettes accumulate carotenoids in the grain^30–32^. The endosperm of GR1 and GR2 is golden in color^30,31^, compared with the white endosperm observed in most conventional rice varieties. Consumption of GR1 and GR2 is predicted to have a positive nutritional impact, especially in regions where rice is the major food source and Vitamin A deficiency is prevalent^33^. Using the Golden Gate Assembly method^34^, we generated a carotenoid cassette based on the published sequence of the GR2 cassette^30^. To reduce the size of the insert, the selectable marker gene and the T-DNA border sequences were not included in our modified cassette. The final 5.2 kb carotenoid cassette (Fig. 1a, Supplementary Data 1) consists of the coding sequences of the two carotenoid biosynthetic genes *SSU-crtI* and *ZmPsy*^30^, both driven by the endosperm-specific glutelin promoter^30^ isolated from Kitaake rice. *SSU-crtI* is a functional fusion of the DNA encoding the chloroplast transit peptide from the pea RUBISCO small subunit and the *Erwinia uredovora* carotenoid desaturase, whereas *ZmPsy* encodes a maize phytoene synthase. The *nopaline synthase* (*nos*) terminator (from *Agrobacterium tumefaciens*) was used for transcription termination in both genes.

### Delivery of the carotenoid cassette into rice at genomic targets

We assembled the donor plasmid *pAcc-B* (Fig. 1a, Supplementary Data 1), which contains the 5.2 kb carotenoid cassette. We added homology arms, which consist of 794 bp and 816 bp of Kitaake genomic sequence to the left and right of the Cas9 cleavage site at the gRNA B target (Target B), respectively. The homology arms were included to facilitate the possibility of homology-directed repair (HDR)^35^, a precise repair mechanism. We placed two gRNA B target sequences outside each homology arm on the donor plasmid to further enhance the chance of targeted insertion of the carotenoid cassette sequence, because linearized donor templates have been reported to increase HDR efficiency^36,37^. We hypothesized that these gRNA target sites would facilitate the release of the carotenoid cassette from the circular donor plasmid by Cas9, based on previous reports^38,39^.

We next constructed the CRISPR plasmid *pCam1300-CRISPR-B*, which consists of a *Cas9p* module^40^ with a Poaceae (the plant family of rice and other species) codon-optimized Cas9-coding sequence driven by the maize *Ubiquitin 1* (*Ubi1*) promoter, and a gRNA B module driven by the promoter of the rice small nuclear RNA gene *OsU6*^41^ (Fig. 1b). The *Cas9p* module also includes the *nos* terminator derived from *Agrobacterium*. A hygromycin resistance selectable marker gene is present on the backbone of *pCam1300-CRISPR-B*, which allows for subsequent selection of rice transformants carrying the *Cas9-gRNA* module using the herbicide hygromycin.

Equal mass of the donor plasmid *pAcc-B* and the CRISPR plasmid *pCam1300-CRISPR-B* were mixed and delivered by particle bombardment (Fig. 1c). We bombarded one hundred Kitaake rice embryogenic calli, and applied hygromycin to select for calli transformed with *pCam1300-CRISPR-B*. We regenerated 55 hygromycin-resistant plants (T0 generation).

### Insertion of the carotenoid cassette occurred at Target B

**Fig. 2:**
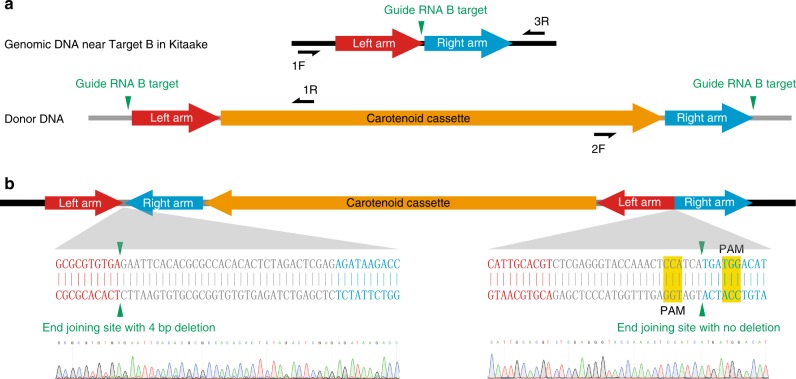
Molecular characterization of the carotenoid cassette at Target B. **a** Diagrams showing the genomic region near Target B in Kitaake rice and the donor DNA. Gray lines represent plasmid backbone DNA while black lines represent Kitaake genomic DNA. The vertical green triangles mark the positions of the guide RNA B target sites. **b** Diagram of the inserted carotenoid cassette at Target B in T0 plants #11, 16, 17, 24, 28, 48, and 50. The junction sequences in all the seven plants are identical, as shown in the diagram. For convenience, only the sequencing chromatograms for T0 #48 are shown. The protospacer adjacent motif (PAM) of the original guide RNA B targets are highlighted in yellow.

We genotyped the 55 T0 individuals by PCR using primers 1F and 1R to check whether carotenoid cassette was inserted at Target B through HDR (Fig. 2a, Supplementary Fig. 3a). We observed a PCR band for T0 plant #1 at 2.6 kb, which exceeds the size of the predicted band by 0.8 kb, roughly the size of the left homology arm. We hypothesized that the left junction of this insertion may have occurred through non-homologous end joining (NHEJ), an alternative pathway to repair DSB with a higher frequency compared with HDR^35^. To test this, we performed additional PCR reactions on T0 plant #1 using primer pairs 1F + 1R and 2F + 3R (Supplementary Fig. 3b–c). A 2.6 kb fragment and a 5.2 kb fragment were amplified, respectively. This result suggests that the entire *pAcc-B* donor plasmid was integrated at Target B in T0 plant #1. Amplicons spanning both junctions of the insert were sequenced to confirm the insertion of the donor plasmid (Supplementary Fig. 3d). Because T0 plant #1 was sterile, we could not harvest seeds to further validate the nature of the insertion.

To assess the possibility that a subset of the remaining T0 plants also harbored the insertion of the carotenoid cassette through NHEJ but in the opposite orientation, we genotyped the 55 T0 plants using primers 1F and 2F (Supplementary Fig. 4a). These primers amplified a 2.3 kb band in seven T0 plants (T0 #11, #16, #17, #24, #28, #48, and #50). Both insertion junctions in these seven plants were confirmed by additional PCR reactions using primer pairs 1F + 2F and 1R + 3R (Supplementary Fig. 4b). Based on these results, we predicted that the donor DNA in between the two gRNA B targets was inserted at Target B through NHEJ in these seven T0 plants (Supplementary Fig. 4b). By sequencing these amplicons, we found that the junctions of the inserts in these seven T0 plants are identical (Fig. 2b). The identity of the junctions suggests that these seven T0 plants are likely clonal derivatives of a single independent insertion, which we subsequently confirmed (see below).

**Fig. 3:**
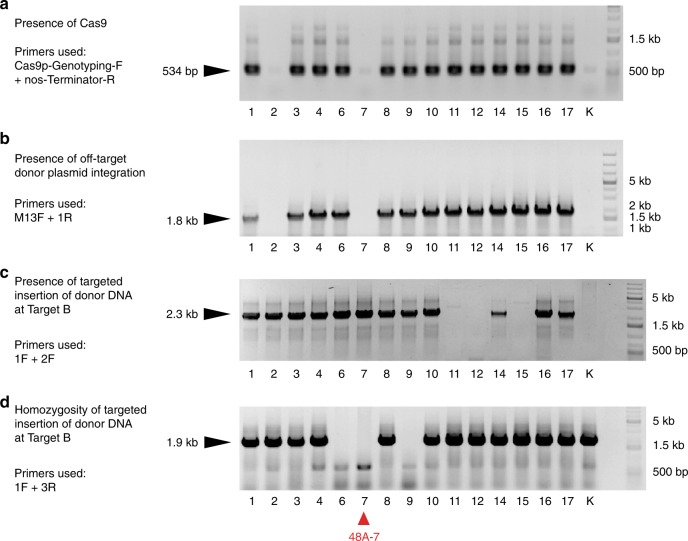
Genetic segregation of the progeny of T0-48A. Genotyping the T1 progeny of T0-48A. The purpose of each PCR experiment and the genotyping primers used are shown to the left for each gel panel. **a** Primers Cas9p-Genotyping-F and nos-Terminator-R amplify a 534 bp DNA fragment in plants with the *Cas9* module. **b** Primers M13F and 1R amplify a 1.8 kb DNA fragment in plants with the off-target insertion of the *pAcc-B* donor plasmid. **c** Primers 1F and 2F amplify a 2.3 kb DNA fragment in plants with the carotenoid cassette inserted at Target B. **d** Primers 1F and 3R amplify a 1.9 kb genomic DNA fragment in plants unless the carotenoid cassette at Target B is homozygous. The positions of the primers used are illustrated in Figs. 1a, b, 2a. Kitaake (K) was used as the negative control. The red triangle at the bottom marks 48A-7, which is free of the co-integrated CRISPR and donor plasmids. Source data are provided as a Source Data file.

We performed genetic segregation analysis of the T1 generation to obtain rice plants homozygous for the carotenoid cassette at Target B that lack the *Cas9-gRNA* module. We genotyped the progeny of 48A (tiller A from T0 #48) for *Cas9* using primers Cas9p-Genotyping-F and nos-Terminator-R located in the *Cas9p* module (Fig. 1b). In parallel, to detect any potential off-target integration of the donor plasmid during particle bombardment in T0 #48, we performed PCR using the donor backbone-specific primer M13F, and the carotenoid cassette-specific primer 1R (Fig. 1a). In the T1 population, the presence of the *Cas9-gRNA* module and the backbone of *pAcc-B* are linked (Fig. 3), suggesting that *pAcc-B* and *pCam1300-CRISPR-B* co-integrated in the genome adjacent to each other in plant T0 #48. This result is consistent with the previously reported observation that multiple plasmids frequently co-integrate when delivered through particle bombardment^42^. We next screened the same T1 population for individuals homozygous for the carotenoid cassette at Target B using primers 1F and 3R (Fig. 2a). From these genetic analyses, we identified T1 individual 48A-7 as being homozygous for the inserted carotenoid cassette at Target B and free of the co-integrated CRISPR and donor plasmids (Fig. 3).

To examine whether the full-length carotenoid cassette was inserted at Target B in 48A-7, we performed PCR using primers 1F and 3R (Fig. 2a) with extended elongation time. A fragment with the expected size of 8.8 kb was amplified (Supplementary Fig. 5a), indicating that the insert in T0 plant #48 at Target B consists of the full-length carotenoid cassette and both homology arms from the donor plasmid. Consistently, a Southern blot assay on the genomic DNA extracted from 48A-7 supports the presence of a single-copy insertion of the full-length carotenoid cassette and the homology arms at Target B (Supplementary Fig. 5b–d). We also carried out the whole-genome sequencing of 48A-7 and identified all the sequencing reads (151 bases in length each) that fully or partially match with the sequence of the donor plasmid *pAcc-B*. We tiled up these reads and reconstructed the sequence of the insert, which is consistent with the sequence of the donor DNA and the Sanger sequencing of the junction ends described in Fig. 2b. We did not detect any DNA sequence of the *pAcc-B* donor plasmid in the genome of 48A-7 besides Target B. Together, these results suggest that plant 48A-7 carries a single copy of the full-length carotenoid cassette at Target B.

To assess the occurrence of off-target mutations caused by Cas9 in the process, we further analyzed the whole-genome sequencing result for 48A-7. We used Cas-OFFinder^43^ to predict potential Cas9 off-target sites in the KitaakeX genome^28^ and identified ten candidate sites (Supplementary Table 3). Sequence analysis indicates that none of the ten predicted off-target sites is mutated in plant 48A-7 (Supplementary Table 3 and Supplementary Data 2). This is consistent with the previously reported absence of mutations at predicted Cas9 off-target sites in rice plants edited with CRISPR-Cas9^44^. Together, these results indicate that DNA cleavage by Cas9 is highly specific to Target B in our experiment.

### Rice plant 48A-7 accumulates β-carotene in the seed

**Fig. 4:**
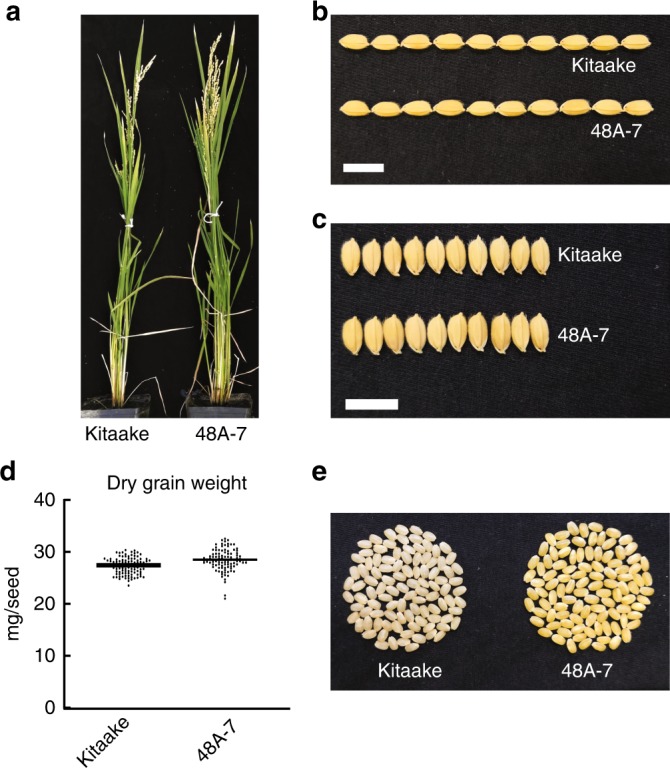
Trait assessment of the homozygous carotenoid-enriched rice line 48A-7. **a** Morphology of the 70-day-old Kitaake and 48A-7 plants. **b** Grain length comparison between Kitaake and the progeny of 48A-7. **c** Grain width comparison between Kitaake and the progeny of 48A-7. **d** Dry grain weight of randomly picked seeds from Kitaake and the progeny of 48A-7 (*n* = 100). Horizontal bars represent the mean value. **e** Picture of 100 randomly picked dehusked seeds from Kitaake and the progeny of 48A-7. White scale bars represent 1 cm. The source data underlying Fig. 4d are provided as a Source Data file.

Plant 48A-7 resembles the control plant Kitaake in plant stature and grain dimensions (Fig. 4a–d). The dehusked seeds derived from 48A-7 are golden in color, indicating the accumulation of carotenoids in the endosperm (Fig. 4e). Because the major carotenoid in the endosperm of GR2 is β-carotene^30^, we quantified the β-carotene content in the endosperm from 48A-7 using high-performance liquid chromatography (HPLC) (Supplementary Fig. 6). In the dehusked, polished seeds from 48A-7, the β-carotene content was 7.90 ± 0.19 μg g^−1^ dry weight (Supplementary Table 4), while no significant amount of β-carotene was detected in the dehusked, polished Kitaake seeds. The observed β-carotene content in 48A-7 is slightly lower than that of the GR2 transformation event GR2E in *japonica* rice variety Kaybonnet under greenhouse conditions (9.22 μg g^−1^ dry weight)^30^, and comparable to the higher end of the range of β-carotene content measured in field-grown *indica* rice variety PSB Rc82 (1.96–7.31 μg g^−1^ dry weight)^32^. The difference in the β-carotene content observed in these studies may be due to the differences in growth conditions, genomic positional effects, and/or post-harvest decay of the carotenoids^45^, the rate of which varies among cultivars^46^. The difference in endogenous carotenoid metabolic components among cultivars may have also contributed to the difference in the level of β-carotene accumulating in the endosperm^47^. Overall, rice plant 48A-7 accumulates a high level of β-carotene in the endosperm.

### Obtaining homozygous marker-free carotenoid-enriched rice

**Fig. 5:**
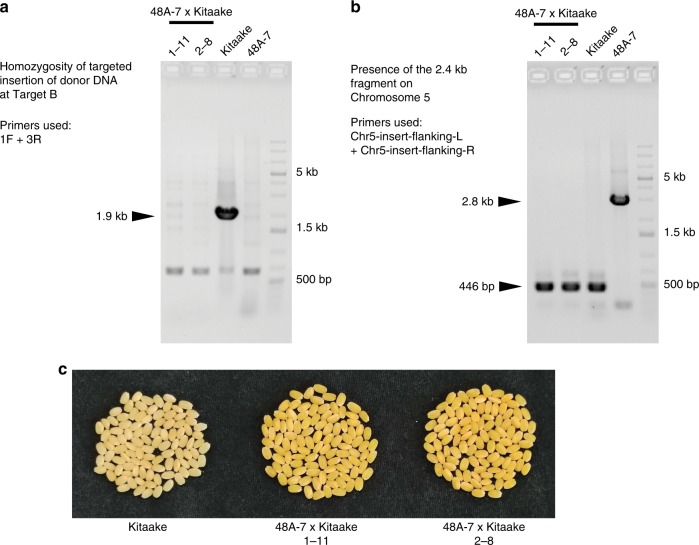
Removal of the 2.4 kb plasmid fragment from 48A-7 by backcross. **a** Checking the homozygosity of the carotenoid cassette inserted at Target B in the F2 individuals 1–11 and 2–8 from the backcross between 48A-7 and Kitaake. PCR primers 1F and 3R anneal to genomic positions flanking Target B, as shown in Fig. 2a, amplifying a 1.9 kb genomic DNA fragment unless the carotenoid cassette at Target B is homozygous. Kitaake and 48A-7 were used as the wild type and homozygous controls, respectively. **b** Detecting the presence of the 2.4 kb CRISPR plasmid fragment on Chromosome 5 by PCR. Primers Chr5-insert-flanking-L and Chr5-insert-flanking-R amplify a 446 bp DNA fragment when the plasmid fragment is absent, or a 2.8 kb DNA fragment when the plasmid fragment is present. Kitaake and 48A-7 were used as the wild type and homozygous controls, respectively. **c** Picture of 100 randomly picked dehusked seeds from Kitaake and the two F2 plants described in (**a**). The source data underlying Fig. 5a, b are provided as a Source Data file.

Analysis of the whole-genome sequence of 48A-7 revealed a fragment of the CRISPR plasmid at an intergenic region on Chromosome 5, the insertion of which is likely caused by the particle bombardment process^48^. We genotyped multiple T0 and T1 plants for this insert by PCR using primers flanking the insertion site. A homozygous 2.4 kb insert was detected in 48A-7 (Supplementary Fig. 7). One copy of this insert was also detected in T0 plants #11, #16, #17, #24, #28, #48, and #50 (Supplementary Fig. 7). This result indicates that these seven T0 plants are most likely derived from a single transformation event, which carries the 2.4 kb fragment resulting from the particle bombardment process. The 2.4 kb insert is absent from T0 plant #1, which suggests that T0 plant #1 resulted from an independent transformation event (Supplementary Fig. 7). To remove the 2.4 kb insert from the homozygous carotenoid-enriched rice, we backcrossed the carotenoid-enriched rice line 48A-7 (maternal) with Kitaake (paternal). The resulting F1 plants were self-pollinated to generate a segregating F2 population. In the F2 generation, we identified two rice plants, 1–11 and 2–8, as being homozygous for the carotenoid cassette at Target B and free of the 2.4 kb insert (Fig. 5a, b). Seeds harvested from both plants are golden in color (Fig. 5c), indicating that both plants accumulate β-carotene in the endosperm. These marker-free carotenoid-enriched rice plants carry homozygous insertion of the carotenoid cassette at the intended genomic target.

### The observed β-carotene is a consequence of the carotenoid cassette at Target B

To confirm that the observed accumulation of β-carotene in the seeds is a consequence of the carotenoid cassette inserted at Target B, we performed a genetic co-segregation analysis. We harvested seeds from 48P-3, a sibling of 48A-7 hemizygous for the insertion at Target B (Supplementary Fig. 8a). A randomly selected tiller from 48P-3 yielded 13 white seeds and 38 golden seeds, which fits the Mendelian ratio of 1:3 for single-site genetic segregation. We randomly germinated eight of the white seeds and eight of the golden seeds and genotyped the seedlings for the presence of the carotenoid cassette at Target B by PCR. The golden seed color co-segregated with the presence of the carotenoid cassette at Target B (Supplementary Fig. 8b). This indicates that the β-carotene in the seeds from 48A-7 results from the targeted insertion of the carotenoid cassette at Target B.

### Targeted insertion of the carotenoid cassette at a second genomic target

To test whether the method of targeted insertion described above can be applied to other chromosomal locations, and to assess the frequency of insertion of the donor DNA, we performed an additional round of co-bombardment experiment at a different target site, Target C. In this experiment, we cultivated each callus separately to prevent clonal propagation. We generated a CRISPR plasmid *pCam1300-CRISPR-C* and a donor plasmid *pAcc-C* (Supplementary Fig. 9 and Supplementary Data 3) and delivered them to rice calli as described in Fig. 1c. We regenerated 16 independent T0 events transformed with the CRISPR plasmid and found that one event, T0 plant #6, carries the insertion of the carotenoid cassette at Target C (based on PCR genotyping and Sanger sequencing of the PCR products (Supplementary Fig. 10)). The insertion occurred through non-homologous end joining, similar to the insertion at Target B observed for T0 plant #48 (Fig. 2b). The overall insertion frequency is 1/16 (6.25%), which represents the number of plants with the on-target insertion of the cassette divided by the total number of transgenic T0 plants carrying the *Cas9-gRNA* module.

## Discussion

Conventional plant genetic engineering methods rely on the insertion of genes encoding desirable agronomic traits at random positions in the genome. This approach can sometimes lead to decreased yields. For example, within GR2-R, one of the independently transformed GR2 events, the GR2 cassette was inserted in the first exon of *OsAux1*, which encodes an auxin transporter essential for plant growth^49^. Homozygous disruption of *OsAux1* by the GR2 cassette in GR2-R causes severe developmental defects and a heavy penalty in yield^49^. Events like this may potentially be reduced with prior knowledge of GSHs within a given genome, and the availability of a reliable tool to insert desired genes at these sites. In this study, we demonstrate the feasibility of this strategy by performing targeted insertion of a carotenoid cassette (consisting of two transcription units) at two separate GSHs in rice. This approach could potentially be applied to any crop species with an established transformation protocol, thus offering a promising tool for plant research and for the genetic improvement of crop plants.

A GSH should accommodate transgene without incurring any undesired trait in the resulting transgenic plant. We based our selection of GSHs in rice on the absence of effects on morphology incurred by mutations at these sites in a rice mutant collection^25,26^. One limitation of this approach is that these selection criteria do not consider the expression level of the transgenes inserted at these sites. To identify GSHs that express transgenes at desired levels, a population of independent transgenic events, each carrying a reporter gene (such as *GFP*) at a distinct insertion site, can be generated and screened. The homozygous transgenic lines expressing the reporter gene without exhibiting unfavorable agronomic traits carry the reproter gene insert at GSHs. Such a screen would also advance our knowledge of the effects of a particular genomic position on gene expression.

For optimal genetic improvement, it is often desirable to combine multiple transgenes located at different loci to achieve heritable stacked traits in a specific cultivar^50^. Although the advent of marker-assisted selection has improved the accuracy and efficiency of the breeding process, stacking transgenes by conventional breeding remains challenging because traits at different loci segregate independently in the progeny^51^. In contrast, with targeted gene insertion as reported in this study, genes encoding multiple traits genes can be stacked at a single genetic locus, which would simplify the downstream breeding efforts.

Further research will be directed at assessing if the presence of the homology arms in the donor plasmid enhances the insertion frequency of large DNA fragments by NHEJ in rice. We will also assess if the removal of the two gRNA target sites from the donor plasmid would facilitate HDR-based gene insertion in rice. It would also be informative to determine whether certain gRNAs can facilitate large DNA insertions during the DNA-repair process as has been demonstrated for human primary T cells^52^.

## Methods

### Plant transformation and growth conditions

Kitaake, a photoperiod-insensitive cultivar of *japonica* rice (*Oryza sativa* sp. *japonica*) with a short generation time, was used in all experiments^24^. For germination, seeds were dehusked and sterilized by incubation in 30% bleach for 15 min with shaking. The residual bleach was washed away with an equal volume of sterilized water for three times and the seeds were germinated on Murashige and Skoog (MS) media containing 1% sucrose and 0.3% Phytagel (Caisson Labs, Smithfield, UT) (pH 5.7) in a growth chamber with the temperature set to 28 °C and a 13 h light/11 h dark regime. One-week-old rice seedlings were transferred to an 80/20 (sand/peat) soil mixture in an environmentally controlled greenhouse with the temperature set to ~28–30 °C and humidity to 75–85% with a 14 h light/10 h dark regime for continued growth until mature. Panicles were harvested and dried at 60 °C for 7 days for long-term storage.

For particle bombardment, Kitaake seeds were sterilized and germinated on the MSD medium (MS with 3% sucrose, 2 mg L^−1^ 2,4-D and 1.2% Agar, pH 5.7) under 28 °C in the dark for 7 days for initial calli induction. Emerging scutella were detached from the seedlings and transferred to fresh MSD medium for continued induction of calli for one month with medium replacement every 10 days. Before bombardment, calli were transferred to the osmotic medium (MS with 3% sucrose, 4.5% mannitol, 4.5% sorbitol, 5 mg L^−1^ 2,4-D and 0.35% Phytagel (Caisson Labs, Smithfield, UT)) for osmotic treatment for 4 h. The bombardment was performed using the PDS-1000/He particle delivery system (Biorad, Hercules, CA) according to the user’s manual. Donor and CRISPR plasmids were pre-mixed in a 1:1 mass ratio before coated onto the gold particles. Each plate of calli was bombarded twice with 1.0 µm gold particles coated with the plasmids at 900–1100 psi with a 6 cm flying distance. After bombardment, the calli were kept on osmotic medium in the dark at 28 °C overnight and then transferred to the MSD medium for recovery at 28 °C for 5 days. Selection and regeneration were performed at 28 °C with a 13 h light/11 h dark regime. For selection, calli were cultured on the MSDH80 medium (MSD with 80 mg L^−1^ Hygromycin B (A.G. Scientific, San Diego, CA)) for 5 weeks. Over this period, calli were moved to fresh MSDH80 medium every 10 days. Calli were regenerated on the MSRH40 medium (MS with 3% sucrose, 0.5 mg L^−1^ NAA, 3 mg L^−1^ BAP, 40 mg L^−1^ Hygromycin B and 1.2% Agar, pH 5.7) for 6 weeks. The regenerated T0 seedlings were transferred to MS medium for rooting for 2 weeks before they were moved to the greenhouse.

### Plasmid construction

A modular CRISPR-Cas9 toolbox system^53^ was used to construct the CRISPR plasmids. Briefly, gRNAs were designed using the CRISPR-PLANT platform^27^. For each gRNA designed, a pair of synthesized oligonucleotides, named Target-A/B/C/D1/D2/E1/E2 -gRNA F and R, were annealed to form a dimer with overhangs at both ends. Each dimer was ligated with the *Bsm*BI-digested plasmid *pYPQ141c* (Addgene) to generate an entry clone with the full-length gRNA. Three-way recombination among *pYPQ167* (Addgene, with the *Cas9p* coding sequence), *pYPQ141c* (Addgene, with the gRNA module) and the destination vector backbone was performed using LR Clonase II (Invitrogen, Carlsbad, CA) at room temperature for 16 h. For the protoplast T7E1 assay, the transient expression vector *pAHC17*^54^ was used as the destination vector. For calli transformation, *pCambia1300* (Cambia, Canberra, Australia) was used as the destination vector. Both destination vectors contain the constitutive maize *Ubi1*^54^ promoter to drive *Cas9* in the final constructs. The *pAcc* starter vector was created by digesting *pBluescript II SK (-)* (Addgene) with *Kpn*I and *Xba*I and ligating the digestion product with an oligodimer formed between primers pAcc-Engineer-F and pAcc-Engineer-R. To construct the donor plasmid, the carotenoid cassette was assembled by ligating four PCR fragments and the *pAcc* plasmid backbone. The names of the eight PCR primers used to generate the four fragments are Cassette-AF/AR/BF/BR/CF/CR/DF/DR. An oligodimer of gRNA target nucleotide sequence was ligated into the donor plasmid on each side of the carotenoid cassette using *Bbs*I and *Bsm*BI, respectively. The names of these primers are Target-B/C-PAM-F/R. The left and right homology arms were PCR-amplified and ligated into the donor plasmid by *Kpn*I and *Xba*I, respectively. The names of the primers used to amplify the homology arms are B/C-Left/Right Arm-F/R. All plasmids were validated by Sanger sequencing (Quintara Biosciences, San Francisco, CA) and by checking the electrophoresis patterns after restriction digestion with the Fast Digest enzymes (Thermo Fisher, Waltham, MA). The sequences of all primers can be found in Supplementary Table 2.

### Plant DNA isolation and genotyping

Genomic DNA was isolated from rice leaf tissue using the cetyltrimethylammonium bromide (CTAB)-chloroform-based method^26^. For each T0 plant, leaf segments from all tillers were harvested and pooled. For all other plants, a single leaf segment was harvested. PCR genotyping was performed using the DreamTaq (Thermo Fisher, Waltham, MA). The GeneRuler 1 kb DNA Ladder (Thermo Fisher, Waltham, MA) was used in all DNA electrophoresis experiments. The Sanger sequencing service was provided by Quintara Biosciences (San Francisco, CA). The sequences of all primers can be found in Supplementary Table 2.

### Southern blotting

Five micrograms of rice genomic DNA was digested overnight using the restriction enzyme *BamH*I (New England Biolabs, Ipswich, MA) for 16 hours at 37 °C. Digested DNA was recovered through ethanol precipitation and split into two equal portions and was run on two duplicate 0.7% agarose gels in parallel. One gel was subject to ethidium bromide staining to determine the completeness of the restriction digestion. The other gel was subject to Southern Blotting with the following procedure. Depurination was performed by rocking the gel in 100 mL of 125 mM HCl for 15 min at room temperature. Denaturation was performed by rocking the gel in 400 mL of the Denaturation Buffer (0.5 M NaOH, 1.5 M NaCl) at room temperature for 1 h. After denaturation, the gel was neutralized in the Neutralization Buffer (1.5 M NaCl, 0,5 M Tris-HCl, pH 7.5) for 30 min with shaking. Overnight blotting of the denatured DNA from the gel onto the Hybond-NX (GE Healthcare, Chicago, IL) membrane was performed in 10× SSC Solution (1.5 M NaCl, 150 mM trisodium citrate, pH 7.0). Transferred DNA was crosslinked to the membrane with a UV crosslinker with the energy set to 70000 µJ cm^−2^. Pre-hybridization treatment of the membrane was performed in a hybridization tube using the DIG Esay-Hyb Buffer (MilliporeSigma, Burlington, MA) at 42 °C with rotation for 30 min. The hybridization probe was labeled with DIG using the DIG DNA Labeling Kit (MilliporeSigma, Burlington, MA), and denatured by incubating at 95 °C for 5 min and immediately chilling on ice. The denatured probe was added to the above-mentioned hybridization tube with the membrane and the DIG Esay-Hyb Buffer. Hybridization was performed at 42 °C for 24 h with rotation. The membrane was washed in 2× SSC (0.3 M NaCl, 30 mM trisodium citrate, pH 7.0) with 0.1% SDS twice at room temperature, 5 minutes each. The membrane was then washed in 0.1× SSC (0.3 M NaCl, 30 mM trisodium citrate, pH 7.0) with 0.1% SDS twice at 65 °C, 15 min each. After washing, the blot was processed using the DIG Luminescent Detection Kit (MilliporeSigma, Burlington, MA). The Chemiluminescent signal was detected with the ChemiDoc XRS (Biorad, Hercules, CA).

### Rice protoplast transformation and the T7E1 assay

Rice protoplasts were prepared from 10-day-old Kitaake seedlings^29,55^. Rice leaf tissue was cut into 0.5-mm-long pieces using a razor blade. Leaf pieces were incubated in the Enzyme Solution (20 mM MES pH 5.7, 10 mM KCl, 0.6 M mannitol, 1.5% Cellulase Onozuka R-10 (RPI Research Products, Mount Prospect, IL), 0.75% Macerozyme R-10 (RPI Research Products, Mount Prospect, IL), 0.1% BSA) in the dark at 25 °C in a sterile Erlenmeyer flask with gentle shaking for 6 hours. An equal volume of the W5 solution (154 mM NaCl, 125 mM CaCl_2_, 5 mM KCl, 2 mM MES pH 5.7) was added to the digest, and the released protoplasts were collected by filtering the digest through a 40-µm nylon mesh. The cells were spun down at 250 g for 3 min at room temperature, and the pellet was washed three times with the W5 solution. The protoplasts were re-suspended in the Mmg Solution (0.4 M Mannitol, 15 mM MgCl_2_, 4 mM MES pH 5.7) to reach 2.5 × 10^6^ cells mL^−1^. To initiate transformation, 200 µL of the polyethylene glycol (PEG) Solution (40% PEG 4000, 0.2 M Mannitol, 100 mM CaCl_2_) was mixed with 200 µL cell resuspension in an Eppendorf tube in the presence of the CRISPR plasmid DNA at a final concentration of 10 ng µL^−1^. The transformation process continued in the dark at 25 °C for 20 min. The transformation was terminated by adding 800 µL of the W5 Solution to the cell resuspension and mixing by inverting the tube. Cells were spun down at 250 g for 3 min at room temperature and the supernatant was discarded. Cells from each tube were resuspended in 2 mL of the WI Solution (0.5 M Mannitol, 20 mM KCl, 4 mM MES pH 5.7, 25 µg mL^−1^ carbenicillin) and kept in the dark at 25 °C for 70 h. Genomic DNA was then extracted from the protoplast cells using the CTAB-chloroform-based method. For the T7E1 assay, genomic DNA fragments spanning various targets were amplified with the Phusion High-Fidelity DNA Polymerase System (Thermo Fisher, Waltham, MA) using primers Target-A/B/C/D/E-PCR- F and R. The PCR products were heated to 95 °C and ramped down to 25 °C over 14 min evenly to allow heteroduplex formation. T7 endonuclease I (New England Biolabs, Ipswich, MA) digestion was performed for 20 minutes at 37 °C. The digestion product was separated by electrophoresis on a 2% agarose gel. The intensity of the bands was quantified using Image J (National Institute of Health), which was used to calculate the proportion of the digested DNA. The sequences of all primers can be found in Supplementary Table 2.

### Carotenoid extraction and HPLC analysis

Rice seeds were dried at ambient temperature for 2 weeks after harvest. Dehusked rice grains were polished using sandpaper and ground into flour in liquid nitrogen using mortar and pestle. About 100 mg of rice flour was rehydrated in 200 μL of water prior to carotenoid extraction and 2.5 μg of β-apo-8′-carotenal was added to each sample for estimation of recovery rate. After incubation in the dark at room temperature for 10 min, the rehydrated rice flour was extracted in 1.25 mL methanol with mixing by a vortex and followed by an additional incubation in the dark for 5 min. The methanolic extract was centrifuged at 13,000 × *g* for 5 min and the supernatant was transferred to a new tube. The pellet was re-extracted with 1.5 mL of diethyl ether twice. The diethyl ether extracts were pooled, combined with the methanolic supernatant, and phase-separated by adding 2 mL of water. The upper phase was saved; the water phase was re-extracted with 1.5 mL of diethyl ether and pooled with the upper phase. The diethyl ether extract was dried under nitrogen gas and the carotenoid residues were resuspended in 320 μL of ethyl acetate. The carotenoid extract (20 μL) was injected on a reverse-phase HPLC and the sample separations were performed^56^. Gradients between two solvents (A) acetonitrile:water:triethylamine (900:99:1, v/v/v) and (B) ethyl acetate were used in HPLC separation, and at a flow rate of 1 mL min-1. The HPLC gradient was 0–5 min, 100–75% A; 5–10 min, 75–30% A; 10–15 min, 30–0% A; 15–16 min, 0–100% A, and 16–17 min, 100% A.

### Analysis of plasmid insertions in 48A-7

For whole-genome sequencing, genomic DNA was isolated from 48A-7 for library construction. The sequencing reaction was performed using the HiSeq 2500 sequencing system (Illumina, San Diego, CA) at the Joint Genome Institute following the manufacturer’s instructions^26^. A BLAST search against the whole-genome sequencing reads was performed for 48A-7 using the donor DNA sequence consisting of the carotenoid cassette plus the two homology arms as the query. Matching reads were identified and overlaid based on overlapping sequences to confirm the structure of the insert at Target B in 48A-7. To examine whether any plasmid DNA is present in the genome at sites other than Target B, we screened all whole-genome sequencing reads for the ones matching the donor plasmid or the CRISPR plasmid and identified the position of these reads in the KitaakeX genome by BLAST. The carotenoid-enriched rice described in this study was generated in the Kitaake genetic background. KitaakeX, a rice genotype whose genome is avialbale^28^, carries an *XA21* transgene in the Kitaake genetic background. We used the KitaakeX reference genome^28^ as a close approximation of the Kitaake genome in this study.

### Predicting potential Cas9 off-target mutation sites in 48A-7

We screened the KitaakeX genome^28^ (https://phytozome.jgi.doe.gov/) for potential off-target mutation sites using the Cas-OFFinder^43^ with default parameters. All potential Cas9 off-target sites with four or fewer nucleotide mismatches compared with the true target of gRNA B were called.

### Analysis of genomic variants between 48A-7 and Kitaake

The paired-end reads for 48A-7 were mapped to the KitaakeX^28^ rice reference genome using the mapping tool Borrow Wheeler Aligner (BWA version 0.5.9) with default parameters^57^. Genomic variants, including single nucleotide variations (SNVs), deletions, and small insertions were called. To call SNVs and small insertions/deletions (<30 bp), we used SAMtools mpileup (-E -C 50 -DS -m 2 -F 0.010638 -d 50000) version 0.1.19+ and bcftools (-bcgv -p 0.989362) for the merged dataset and filtered using vcfutils.pl (-D 50000 -w0 -W0 -10 -20 -40 -e0) from the SAMtools package^58^. The minimum QUAL score was 100 for SNVs. Pindel version 0.2.4w^59^ was run with default parameters using BreakDancer^60^ results as the input. Small insertions/deletions simultaneously called by SAMtools and Pindel were kept; those called only by SAMtools were filtered at a QUAL score of 100, and those only called by Pindel were further filtered with the following criteria: (1) the variation site had a minimum 10 reads, (2) at least 30% of the reads supported the variation. From the variants between 48A-7 and KitaakeX, we subtracted the known variants between Kitaake and KitaakeX^28^. The remaining are the true variants between 48A-7 and its genetic background Kitaake. These genomic variants were compared with the predicted Cas9 off-target sites to evaluate the occurrence of Cas9-induced off-target mutations.

### Reporting summary

Further information on research design is available in the Nature Research Reporting Summary linked to this article.

## Supplementary information

Supplementary Information

Reporting Summary

Description of Additional Supplementary Files

Supplementary Data 1

Supplementary Data 2

Supplementary Data 3

## Data Availability

A reporting summary for this article is available as a Supplementary Information file. Data supporting the findings of this work are available within the paper and its Supplementary Information files. The datasets generated and analyzed during the current study are available from the corresponding author upon request.The whole-genome sequencing data for 48A-7 have been deposited into NCBI’s Sequence Read Archive udern accession number SRP174336. The source data underlying Figs. 3, 4d, 5a–b and Supplementary Figs. 1, 2b, 3a–c, 4, 5a–c, 7, 8, and 10b are provided as a Source Data file.

## Source Data

Source Data
