# Supplementary material for: Marker-free carotenoid-enriched rice generated through targeted gene insertion using CRISPR-Cas9
Source: Nat Commun. 2020 Mar 4;11:1178. doi: 10.1038/s41467-020-14981-y (PMC7055238; doi:10.1038/s41467-020-14981-y)
Supplement: Supplementary file 1 — Supplementary Information [file 41467_2020_14981_MOESM1_ESM.pdf]

Marker-free carotenoid-enriched rice generated through targeted gene insertion using CRISPR-Cas9

Dong *et al.*

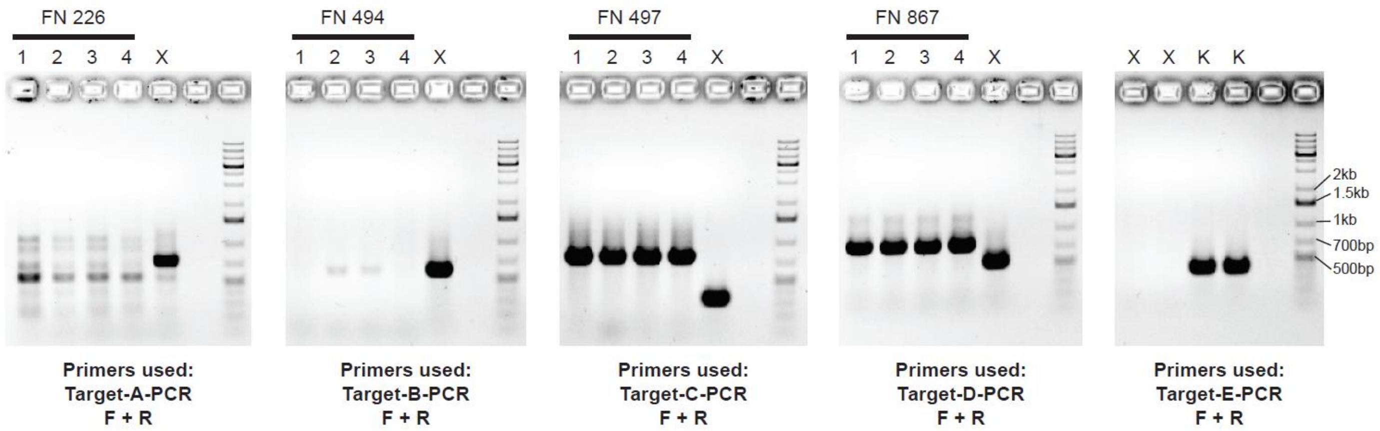

Supplementary Figure 1. Verifying homozygous mutations in the five rice mutants. Homozygous mutations listed in Supplementary Table 1 are verified by PCR in the corresponding rice mutant lines. Each panel shows the genotyping of one mutation site. The primers used are indicated below each panel. The first four panels show the genotyping of the four fast-neutron (FN) mutant lines in Supplementary Table 1. For each mutant line, four siblings were included as biological replicates. KitaakeX (X), the genetic background of these FN mutants, was included as the negative control. The last panel shows the genotyping of two KitaakeX plants at the homozygous *XA21* transgene insertion site. Kitaake (K), the genetic background of KitaakeX (X), was included as the negative control. Source data are provided as a Source Data file.

a

Target Name	Target Sequence
A	CAAGGCACTCAACTACGTGAGG
B	GTGGCGCGTGTGAATTCTGATGG
C	CTACTCCGGAGTCCGGACAGGG
D1	TGGTTTTCGAACATACGGTGGG
D2	GGTGGTTTACAAACATACGATGG
E1	CGGGGAGAAGAACGAGACTCGG
E2	GGAAGACGCCTTCGACAGGTGGG

b

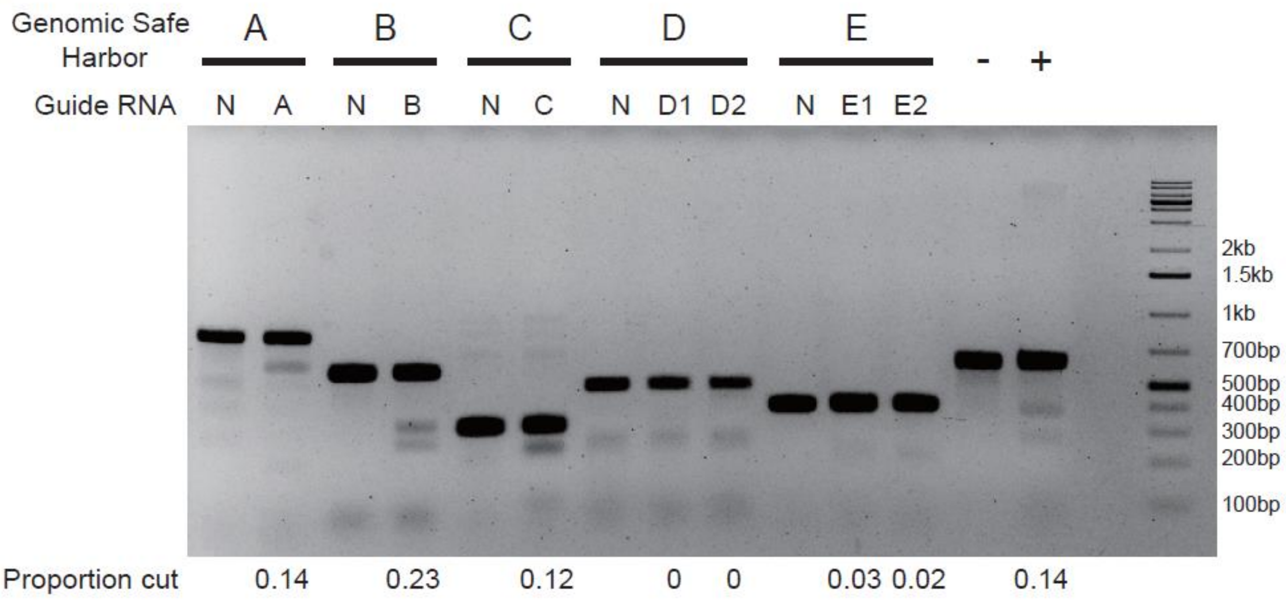

Supplementary Figure 2. Identifying guide RNA targets where Cas9 cleavages efficiently. a, Nucleotide sequences of the seven guide RNA targets. The protospacer adjacent motif (PAM) sequences are colored in red. b, T7 Endonuclease I (T7E1) assay performed using rice protoplasts transiently expressing *Cas9* and each of the guide RNAs shown in (a). Protoplasts transformed with the empty expression vector pAHC17 without the *Cas9-gRNA* module were used as the negative (N) control for each guide RNA tested. DNA template with and without a heterozygous single nucleotide variance was included as the positive (+) and negative (-) T7E1 digestion control, respectively. The numbers below the lanes indicate the proportion of DNA cleaved by T7E1. The source data underlying Supplementary Figure 2b are provided as a Source Data file.

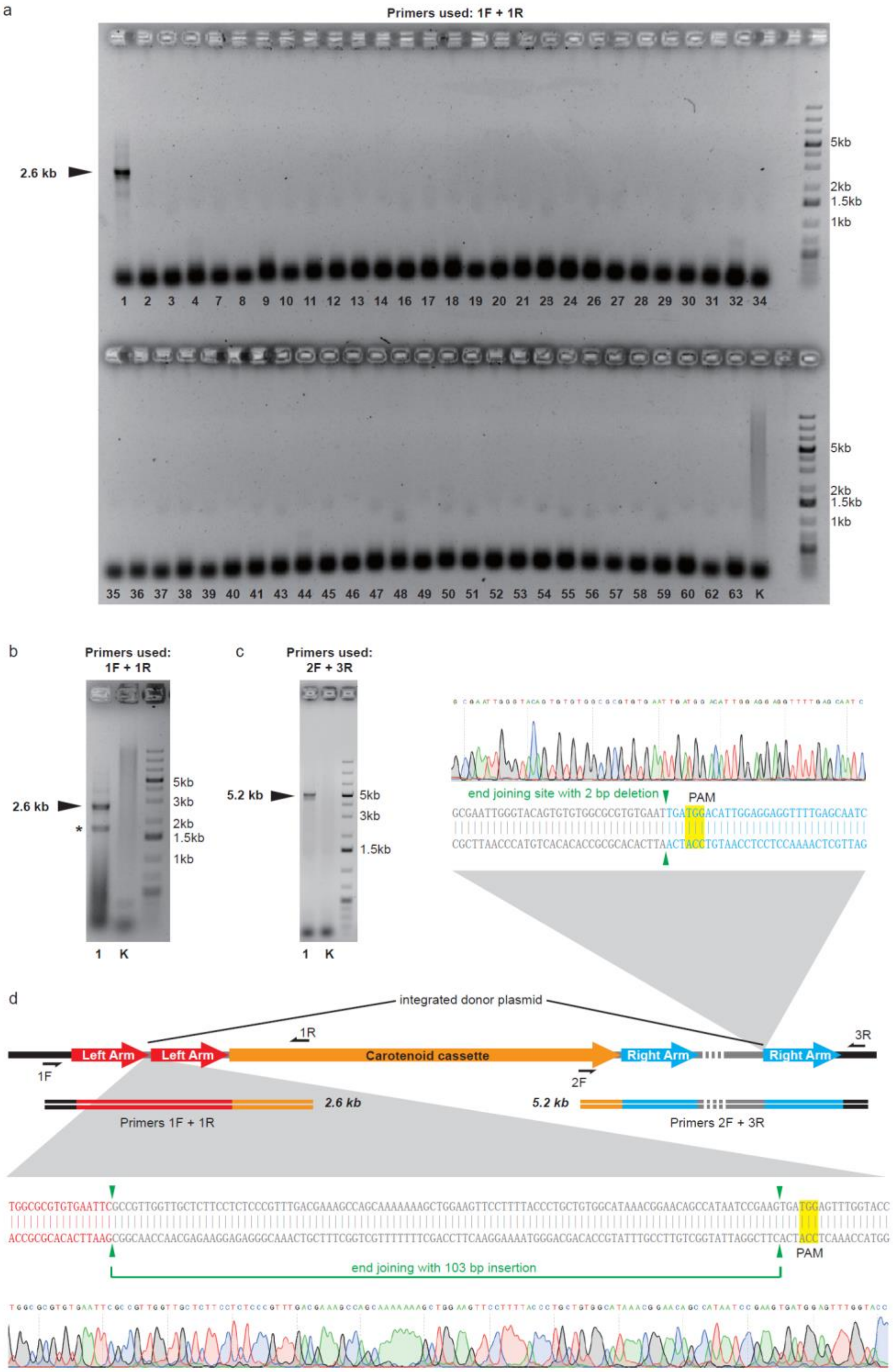

Supplementary Figure 3. The entire donor plasmid integrated at Target B in T0 plant #1. a, Genotyping the 55 T0 plants using primers 1F and 1R. b, Genotyping T0 plant #1 using primers 1F and 1R. The asterisk (*) marks a hypothesized secondary amplification product due to a PCR artifact that eliminates one copy of the left arm. c, Genotyping T0 plant #1 using primers 2F and 3R. Kitaake (K) was used as the negative control in (a)-(c). d, Diagram of the inserted plasmid at Target B in T0 plant #1. The broken grey lines represent the scaffold of the donor plasmid *pAcc-B*. Primers and the corresponding PCR products are illustrated in the diagram. Sequences covering both junction ends are shown with the sequencing chromatograms displayed. The green triangles mark the joining positions. The protospacer adjacent motif (PAM) of the original guide RNA B targets are highlighted in yellow. The source data underlying Supplementary Figure 3a-c are provided as a Source Data file.

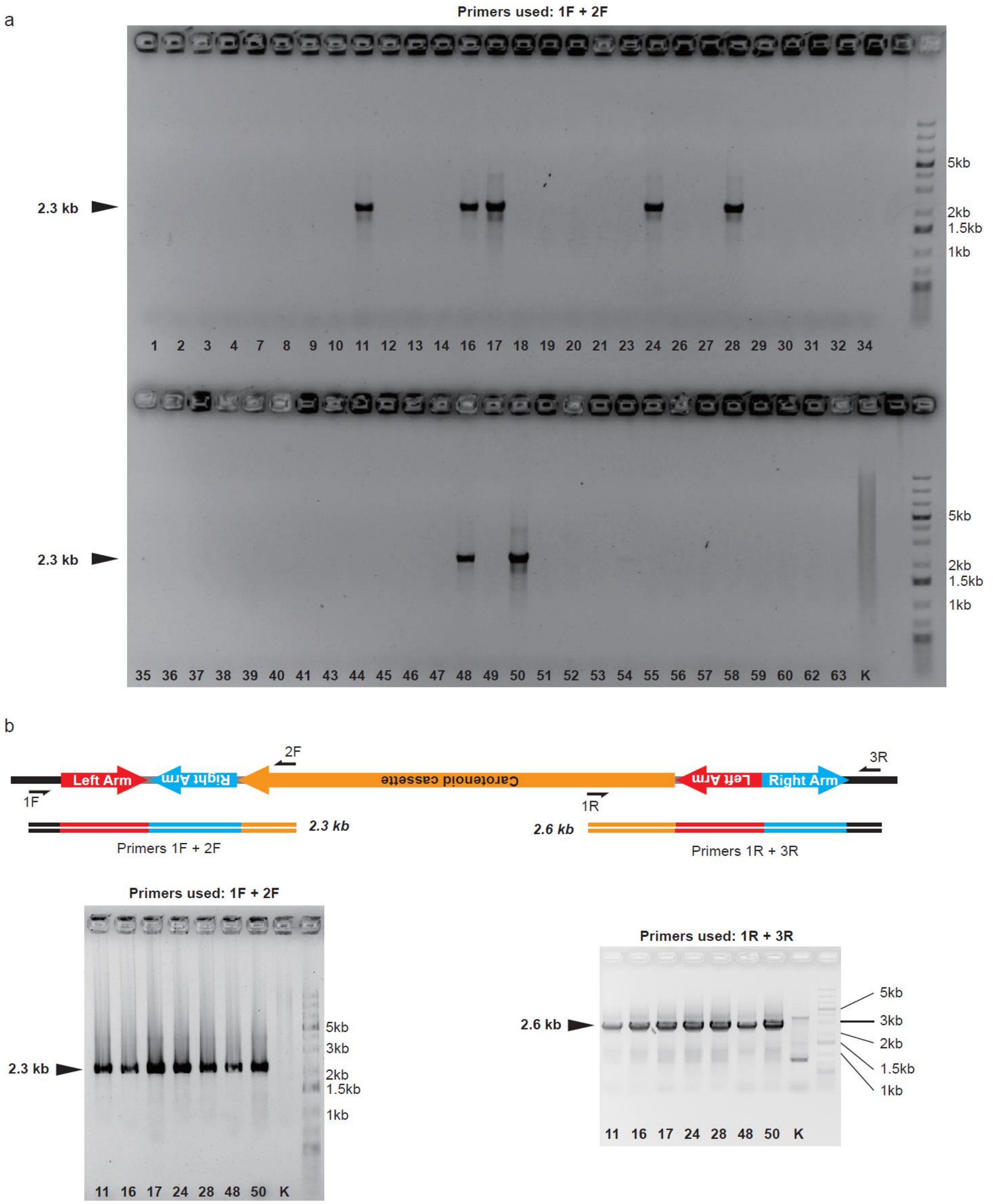

Supplementary Figure 4. The carotenoid cassette was inserted at Target B in seven T0 plants through end joining. a, Genotyping the 55 T0 plants using primers 1F and 2F. b, Genotyping the seven T0s plants that are positive in (a) for both junctions of the insert. A diagram illustrates the hypothesized insertion, the genotyping primers, and the expected PCR products. Gel pictures below the diagram are the genotyping results. Kitaake (K) was used as the negative control in (a) and (b). Source data are provided as a Source Data file.

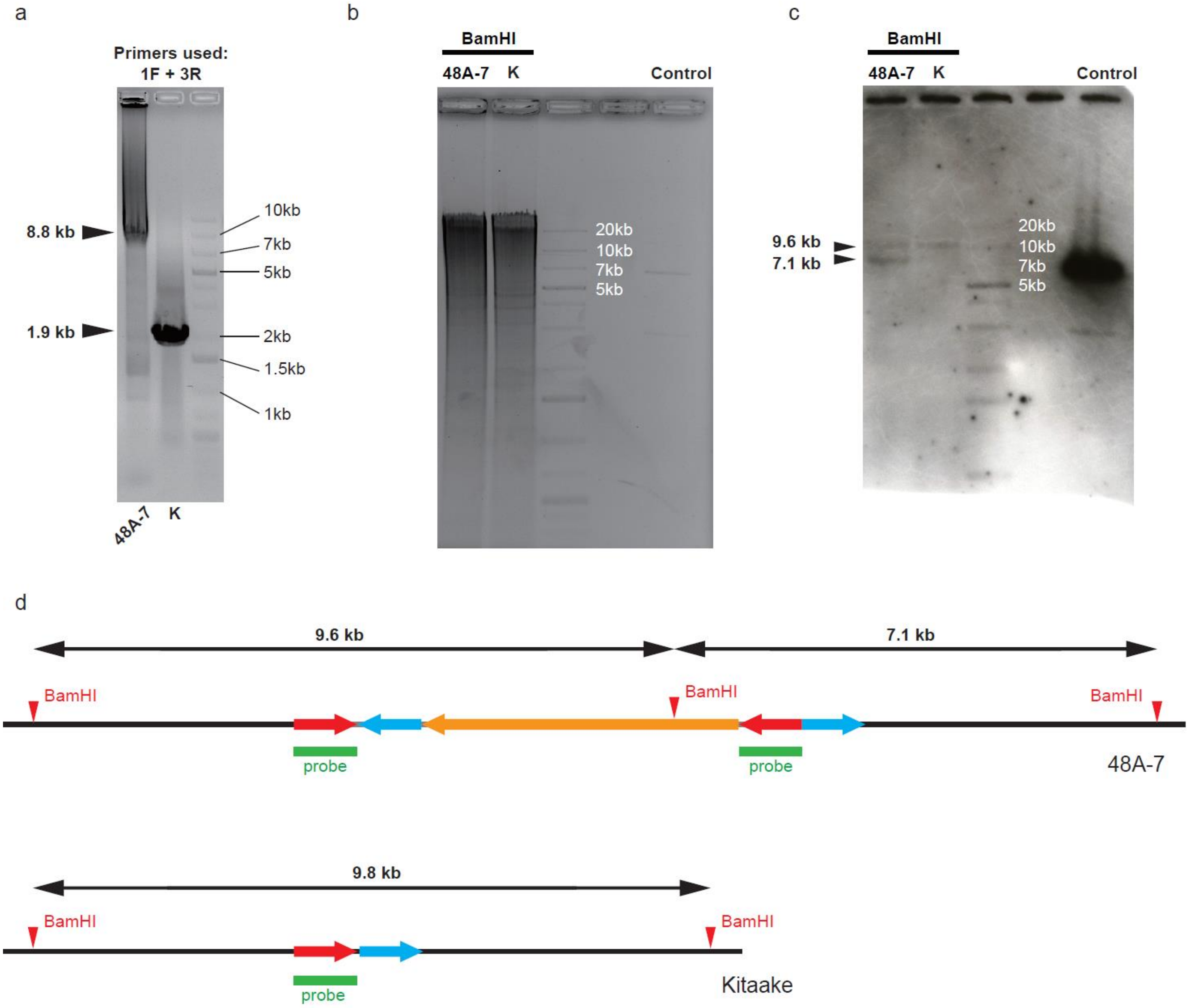

Supplementary Figure 5. Homozygous insertion of the full-length carotenoid cassette at Target B in plant 48A-7. a, Genotyping 48A-7 for the full-length carotenoid cassette at Target B. PCR primers 1F and 3R anneal to genomic positions flanking Target B, as shown in Figure 2a. b, Electrophoresis of BamHI-digested genomic DNA from 48A-7 and Kitaake (K). Linearized donor plasmid *pAcc-B* was used as the control. c, Southern Blot of the gel in (b). DNA ladders are shown on the right side while the sizes of the predicted hybridized fragments in 48A-7 are indicated by arrows on the left. d, Diagram showing the probe used in (c) and the genomic fragments it hybridizes with in 48A-7 (upper) and Kitaake (lower). The source data underlying Supplementary Figure 5a-c are provided as a Source Data file.

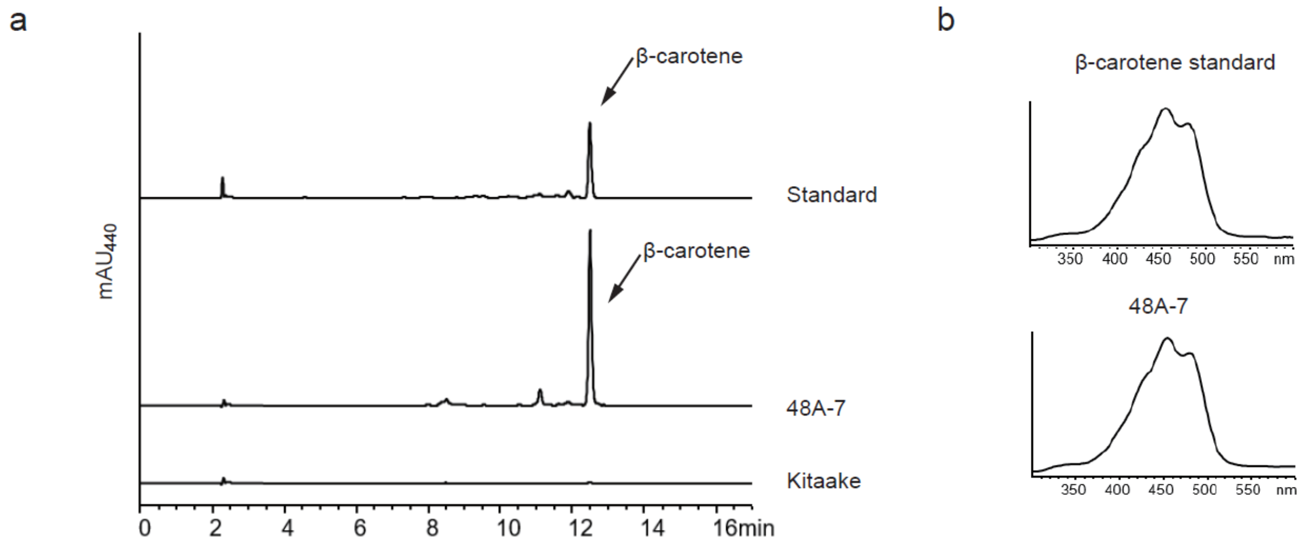

Supplementary Figure 6. Accumulation of β-carotene in seeds from 48A-7. a, High-performance liquid chromatography elution profiles of rice flour prepared from 48A-7 (middle) and Kitaake (lower) and a commercial β-carotene standard as reference (upper). The peak corresponding to β-carotene is indicated by arrows. The x-axis is the retention time in minutes while the y-axis is the absorbance at 440nm. mAU, milli absorbance unit. b, Absorption spectra of the commercial β-carotene standard (upper) and the main peak of 48A-7 (lower), with the x-axis indicating wavelengths and the y-axis indicating absorbance.

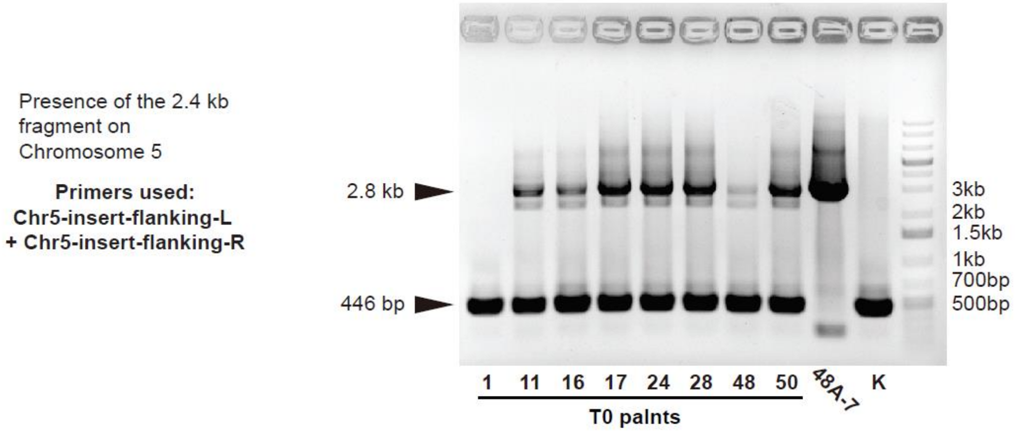

Supplementary Figure 7. Presence of the CRISPR plasmid fragment in 48A-7 and multiple T0 plants. Detecting the insertion of the CRISPR plasmid fragment on Chromosome 5 by PCR. Primers Chr5-insert-flanking-L and Chr5-insert-flanking-R amplify a 446 bp DNA fragment when the plasmid fragment is absent, or a 2.8 kb DNA fragment when the plasmid fragment is present. Kitaake (K) was used as the negative control. Source data are provided as a Source Data file.

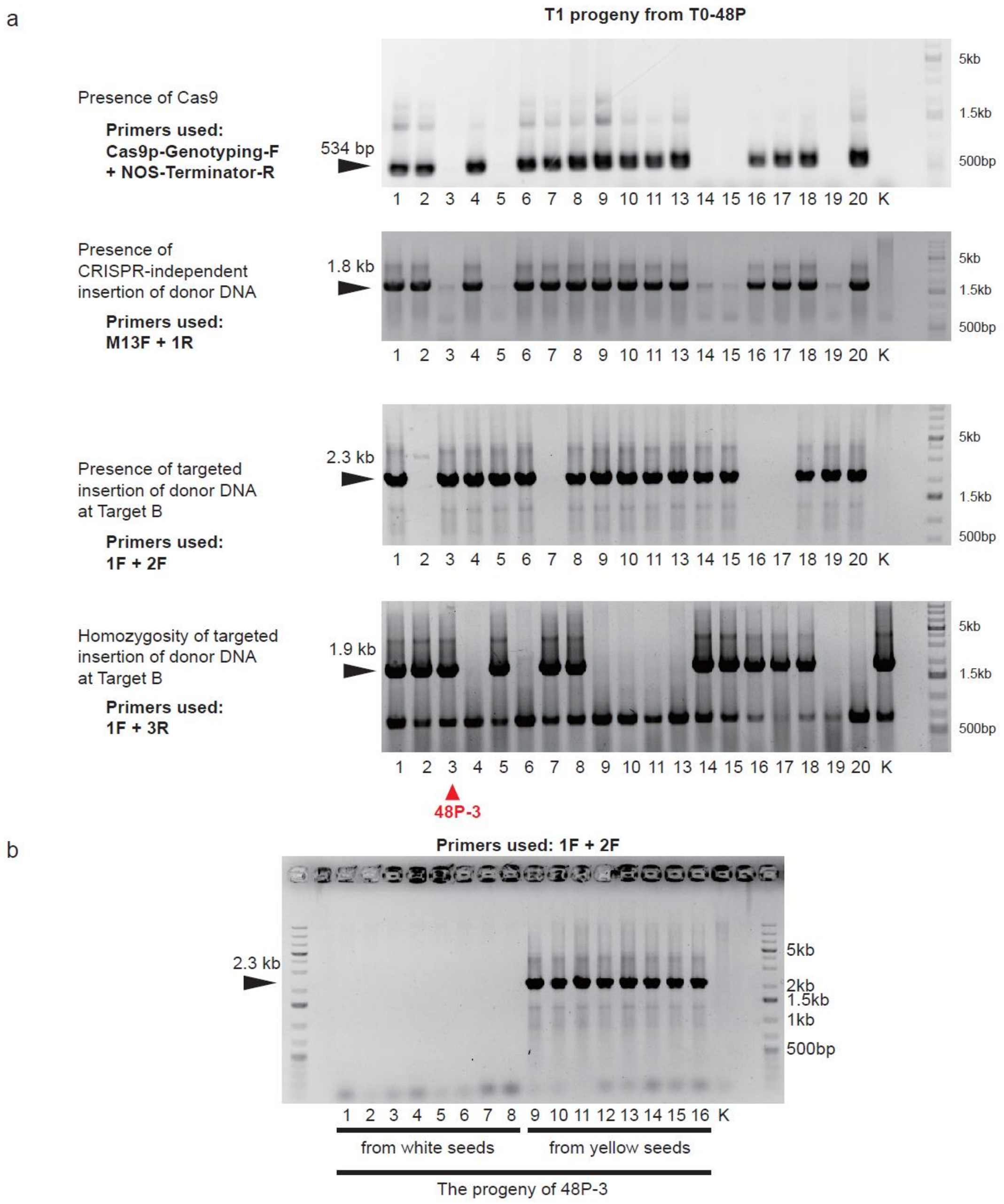

Supplementary Figure 8. The golden seed color co-segregates with the presence of the carotenoid cassette. a, Genotyping T1 progenies derived from T0-48P, a tiller of T0 plant #48. The purpose of each PCR experiment and the genotyping primers used are shown to the left for each gel panel. From top to bottom: Primers Cas9p-Genotyping-F and nos-Terminator-R amplify a 534 bp DNA fragment in plants with the *Cas9* module; Primers M13F and 1R amplify a 1.8 kb DNA fragment in plants with the off-target insertion of the *pAcc-B* donor plasmid; Primers 1F and 2F amplify a 2.3 kb DNA fragment in plants with the carotenoid cassette inserted at Target B; Primers 1F and 3R amplify a 1.9 kb genomic DNA fragment in plants unless the carotenoid cassette at Target B is homozygous. The positions of the primers used are illustrated in Figure 1a, 1b, and Figure 2a. Kitaake (K) was used as the negative control. The red triangle marks 48P-3. b, Genotyping progeny of the T1 individual 48P-3 for the presence of the carotenoid cassette at Target B. Plants #1-8 are derived from white seeds while plants #9-16 are derived from yellow seeds. Kitaake (K) was used as the negative control. Source data are provided as a Source Data file.

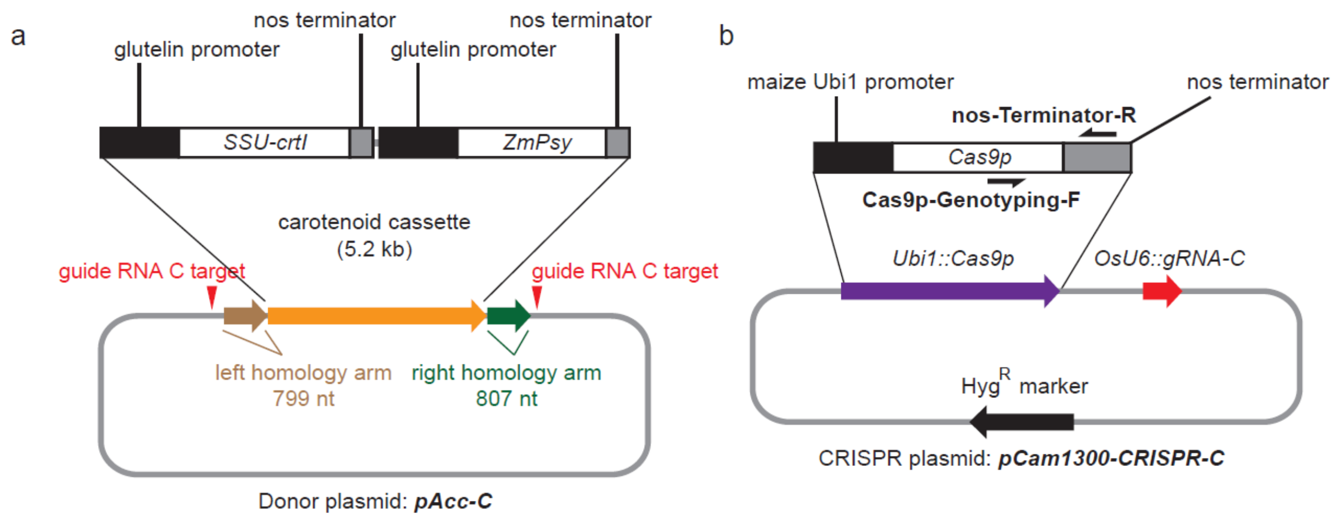

Supplementary Figure 9. Maps of plasmids used for targeted gene insertion at Target C. a, Map of the donor plasmid *pAcc-C* with details of the carotenoid cassette (orange arrow). Brown and green arrows represent the homology arms. The two vertical red triangles mark the two guide RNA C target sites. The nucleotide sequence of the donor plasmid is presented in Supplementary Data 3. b, Map of the CRISPR plasmid *pCam1300-CRISPR-C*. Genes encoding *Cas9p*, *gRNA-C*, and hygromycin resistance (Hyg^R) are represented by purple, red, and black arrows, respectively. The *Cas9p* module is shown in detail. Primers used to genotype *Cas9p* are marked on the map.

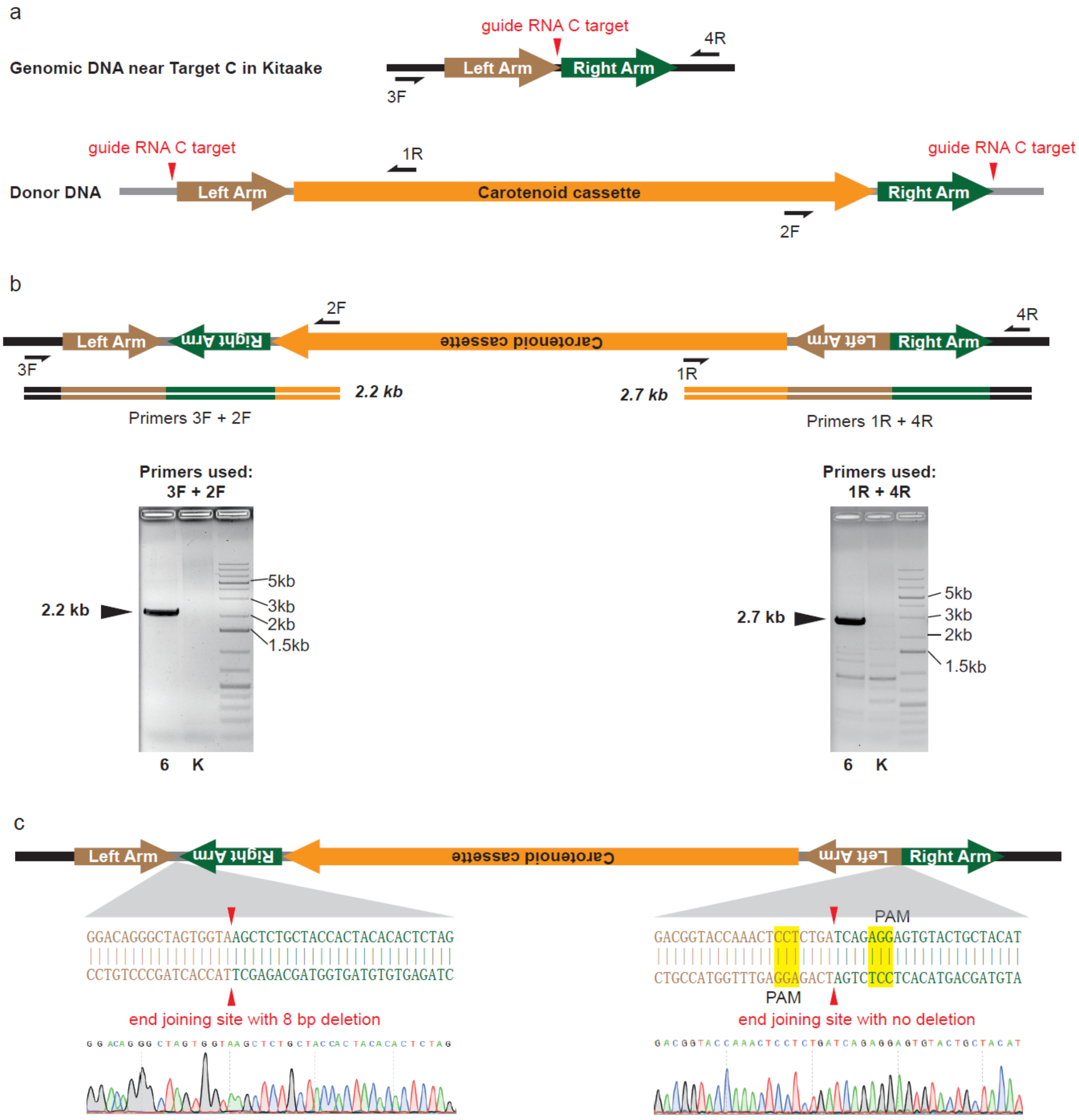

Supplementary Figure 10. Insertion of the carotenoid cassette at Target C. a, Diagrams showing the genomic region near Target C in Kitaake rice and the donor DNA. Grey lines represent plasmid backbone DNA while black lines represent Kitaake genomic DNA. The vertical red triangles mark the positions of the guide RNA C targets. b, Genotyping T0 plant #6 for the insertion of the carotenoid cassette at Target C. A diagram illustrates the insertion, the genotyping primers, and the expected PCR products. Gel pictures below the diagram are the actual genotyping results. Kitaake (K) was used as the negative control. c, Junction border sequences of the carotenoid insert in T0 plant #6. Sanger sequencing chromatograms of the PCR products in (b) are shown. The protospacer adjacent motif (PAM) of the original guide RNA C targets are highlighted in yellow. The source data underlying Supplementary Fgirue 10b are provided as a Source Data file.

Supplementary Table 1. Five fast-neutron rice mutants with wild type morphology and the homozygous mutations they carry.

Mutant name	Genetic background	Type of mutation (homozygous)	Genomic position	Name of candidate genomic safe harbor
FN 226	KitaakeX	Insertion	Chr6:12611821	GSH-A
FN 494	KitaakeX	Translocation	Chr1:42355239	GSH-B
FN 497	KitaakeX	Insertion	Chr1:4898432	GSH-C
FN 867	KitaakeX	Insertion	Chr3:10490206	GSH-D
KitaakeX	Kitaake	Transgene insertion	Chr6:28154158	GSH-E

Note: Genomic positions of the mutations are based on the KitaakeX reference genome. Detailed information on the mutations can be found at https://kitbase.ucdavis.edu. All plants in the first column exhibit Kitaake wild type-like morphology.

Supplementary Table 2. Primers used in this study

Name	Sequence
Target-A-gRNA-F	GTGTGCAAGGCACTCAACTACGTG
Target-A-gRNA-R	AAACCACGTAGTTGAGTGCCTTGC
Target-B-gRNA-F	GTGTGTGGCGCGTGTGAATTCTGA
Target-B-gRNA-R	AAACTCAGAATTCACACGCGCCAC
Target-C-gRNA-F	GTGTGTAGTGGTAGCAGAGCTCAG
Target-C-gRNA-R	AAACCTGAGCTCTGCTACCACTAC
Target-D1-gRNA-F	GTGTGTGGTTTTCGAACATACGGT
Target-D1-gRNA-R	AAACACCGTATGTTCGAAAACCAC
Target-D2-gRNA-F	GTGTGGTGGTTTACAAACATACGA
Target-D2-gRNA-R	AAACTCGTATGTTTGTAAACCACC
Target-E1-gRNA-F	GTGTGCGGGGAGAAGAACGAGACT
Target-E1-gRNA-R	AAACAGTCTCGTTCTTCTCCCCGC
Target-E2-gRNA-F	GTGTGGAAGACGCCTTCGACAGGT
Target-E2-gRNA-R	AAACACCTGTCGAAGGCGTCTTCC
Target-A-PCR-F	GCCTGACAGTGCGTGGTC
Target-A-PCR-R	GCCTCATCGCTCCTCGTGAT
Target-B-PCR-F	GACAGTTGGTGATATGTCAATCACGC
Target-B-PCR-R	TTTGCCGCTTCGATTCGTGT
Target-C-PCR-F	GAATAGCAGAGTCCACGAGACGA
Target-C-PCR-R	TTTAGAGTACGTGGGCACGTCG
Target-D-PCR-F	TTCGGATGTGAACAATACACTGCTAT
Target-D-PCR-R	ACATTAGAATCCATTTCCATAATTAAGGG
Target-E-PCR-F	GGCGACGGCAAACCCGATG
Target-E-PCR-R	GGCCACGCCTCCTGCACTA
Cassette-AF	CGGGGTACCGGTCTCGCTATCGTTAATCATGGTGTAGGCAA
Cassette-AR	GCTCTAGAGGTCTCACATGGGTTGTTGTAGGACTAATGAACTG
Cassette-CF	CGGGGTACCGGTCTCAGATCCGTTAATCATGGTGTAGGCAA
Cassette-CR	GCTCTAGAGGTCTCAGTGTAGTTGTTGTAGGACTAATGAACTG
Cassette-BF	CGGGGTACCGGTCTCACATGAGAATTCGGCTTCCCAAATC
Cassette-BR	GCTCTAGAGGTCTCAGATCGAAGCTTATTAAGGCCCGATC
Cassette-DF	CGGGGTACCGGTCTCTACACTGAATTCGGCTTCCCAAAT
Cassette-DR	GCTCTAGAGGTCTCTCTGGACGATCTAGTAACATAGATGACACC
Target-B-PAM-F	GTGTGTGGCGCGTGTGAATTCTGATGGA
Target-B-PAM-R	AAACTCCATCAGAATTCACACGCGCCAC
Target-C-PAM-F	GTGTGTAGTGGTAGCAGAGCTCAGAGGA
Target-C-PAM-R	AAACTCCTCTGAGCTCTGCTACCACTAC
B-Left-Arm-F	CGGGGTACCCTCGAGACGTGCAATGGAGTGTAATAC
B-Left-Arm-R	CGGGGTACCGAATTCACACGCGCCACAAT
B-Right-Arm-F	TGCTCTAGATGATGGACATTGGAGGAGGTT
B-Right-Arm-R	TGCTCTAGACTCGAGAGATAAGACCAGGTTTGGC
C-Left-Arm-F	CGGGGTACCGTCAGGATTGGTAGGAATTAGC
C-Left-Arm-R	CGGGGTACCAGCTCTGCTACCACTAGCC
C-Right-Arm-F	TGCTCTAGAGAGGAGTGTACTGCTACATGATG
C-Right-Arm-R	TGCTCTAGAAGAGGTAGGACGTTACAGGTGT
1F	ATTGGCAACGCACTGGATTC
2F	GGCAGATCCTGGATGAGATC
1R	GGAAGCCGAATTCTCATGG
3R	CTCATGGAAGGTGCACAACC
3F	AGGACGGAGAAAGTACTGCATAG
4R	AAGAACTCCGAGGTTAAAGCG
M13F	GTAAAACGACGGCCAGT
Cas9p-Genotyping-F	CGAGAACATCATCCACCTCTTCA
nos-Terminator-R	ATCTAGTAACATAGATGACACCGCG
pAcc-Engineer-F	AGTGTGGGTCTTCGAGAAGACCTGTTTGGTACCCCGCTAGTCTAGAGTGTGGAGACGAT TGCGTCTCTGTTTA
pAcc-Engineer-R	CTAGTAAACAGAGACGCAATCGTCTCCACACTCTAGACTAGCGGGGTACCAAACAGGT CTTCTCGAAGACCCACACTGTAC
Chr5-insert-flanking-L	AATAACAGAGAGGCTGAGAGTC
Chr5-insert-flanking-R	GGAGAAGCGTGGGAATAAGAA

Supplementary Table 3. Absence of mutations at the predicted off-target sites in 48A-7.

Chromosome	Position	Sequence	Number of Mismatches	Mutation in 48A-7
Chr1	42355133	GTGGCGCGTGTGAATTCTGATGG	0	/
Chr8	8900142	GTGGgGCGTGTGAAgTgTGAGGG	3	None
Chr1	25553130	GTacCaCGTcTGAATTCTGATGG	4	None
Chr2	34852771	GTGGCGttTcTGAAcTCTGATGG	4	None
Chr3	5583380	GTGGCGtGcGgGAtTTCTGACGG	4	None
Chr3	17714129	GTGGCcCaTaTGAgTTCTGAAGG	4	None
Chr3	35701456	GTttCcCtTGTGAATTCTGATGG	4	None
Chr5	12822698	aTGGCcCaTGTGAgTTCTGAAGG	4	None
Chr6	28102882	GcGGCGCGgGTGAAagCTGAGGG	4	None
Chr9	11295034	GaGGCGCGTGTGgATcCTGcCGG	4	None
Chr10	21956160	GTGatGCGTGTGAATcgTGATGG	4	None

Note: Positions and sequences of the intended insertion target (Target B) and the ten predicted Cas9 off-target sites in the KitaakeX genome. Nucleotides different from the intended target are indicated by lowercase letters and were colored in red. The putative protospacer adjacent motif (PAM) sequences are highlighted in yellow.

Supplementary Table 4. Raw data of the β-carotene quantification in rice line 48A-7.

Rice sample	Area of the β-carotene peak on HPLC chromatogram	Peak area conversion to β-carotene quantity (μg) based on a standard curve	Grain weight (g)	μg β-carotene /g flour	Average (μg g^{-1})	Standard deviation (μg g^{-1})
48A-7-1	662.6	0.766	0.0989	7.74	7.90	0.19
48A-7-2	696.5	0.815	0.1038	7.85		
48A-7-3	700.1	0.838	0.1033	8.12		

Note: Rice seeds harvested from plant 48A-7 were split into three portions, polished, ground to rice flour, and measured separately as technical replicates.
