# Supplementary material for: Marker-free carotenoid-enriched rice generated through targeted gene insertion using CRISPR-Cas9
Source: Nat Commun. 2020 Mar 4;11:1178. doi: 10.1038/s41467-020-14981-y (PMC7055238; doi:10.1038/s41467-020-14981-y)
Supplement: Supplementary file 3 — Description of Additional Supplementary Files [file 41467_2020_14981_MOESM3_ESM.docx]

**Description of Additional Supplementary Files**

File name: Supplementary Data 1
Description: The full sequence of the donor plasmid *pAcc-B*. The full sequence and various sections of the donor plasmid *pAcc-B* are displayed. A diagram of the *pAcc-B* plasmid is shown as Figure 1a.

File name: Supplementary Data 2
Description: Genomic variantions between 48A-7 and KitaakeX.

File name: Supplementary Data 3
Description: The full sequence of the donor plasmid *pAcc-C*. The full sequence and various sections of the donor plasmid *pAcc-C* are displayed. A diagram of the *pAcc*-C plasmid is shown in Supplementary Figure 9.
